# Supplementary material for: Antioxidant Potential of Spray- and Freeze-Dried Extract from Oregano Processing Wastes, Using an Optimized Ultrasound-Assisted Method
Source: Foods. 2023 Jul 7;12(13):2628. doi: 10.3390/foods12132628 (PMC10341308; doi:10.3390/foods12132628)
Supplement: Supplementary file 1 [file foods-12-02628-s001.zip › foods-2487127-supplementary.pdf]

Supplamentary material

ANOVA tables for optimization experiments

Table S1. Analysis of Variance for FRAP

Source	DF	Adj SS	Adj MS	F-Value	P-Value
Model	9	0.040373	0.004486	4.19	0.018
Linear	3	0.033435	0.011145	10.40	0.002
time	1	0.012602	0.012602	11.77	0.006
Ratio S/S	1	0.001823	0.001823	1.70	0.221
Ratio E/W	1	0.019010	0.019010	17.75	0.002
Square	3	0.002681	0.000894	0.83	0.505
time*time	1	0.000838	0.000838	0.78	0.397
Ratio S/S*Ratio S/S	1	0.000838	0.000838	0.78	0.397
Ratio E/W*Ratio E/W	1	0.001218	0.001218	1.14	0.311
2-Way Interaction	3	0.004257	0.001419	1.32	0.320
time*Ratio S/S	1	0.004232	0.004232	3.95	0.075
time*Ratio E/W	1	0.000012	0.000012	0.01	0.916
Ratio S/S*Ratio E/W	1	0.000013	0.000013	0.01	0.916
Error	10	0.010711	0.001071		
Lack-of-Fit	5	0.004316	0.000863	0.67	0.662
Pure Error	5	0.006395	0.001279		
Total	19	0.051084			

Table S2. Analysis of Variance for % antioxidant capacity

Source	DF	Adj SS	Adj MS	F-Value	P-Value
Model	9	161.398	17.9331	4.76	0.011
Linear	3	80.694	26.8979	7.15	0.008
time	1	70.462	70.4617	18.72	0.001
Ratio S/S	1	3.817	3.8174	1.01	0.338
Ratio E/W	1	6.415	6.4147	1.70	0.221
Square	3	71.776	23.9254	6.36	0.011
time*time	1	18.152	18.1517	4.82	0.053
Ratio S/S*Ratio S/S	1	0.430	0.4296	0.11	0.742
Ratio E/W*Ratio E/W	1	4.724	4.7235	1.26	0.289
2-Way Interaction	3	8.928	2.9761	0.79	0.526
time*Ratio S/S	1	2.121	2.1208	0.56	0.470
time*Ratio E/W	1	5.132	5.1317	1.36	0.270
Ratio S/S*Ratio E/W	1	1.676	1.6757	0.45	0.520
Error	10	37.637	3.7637		
Lack-of-Fit	5	19.266	3.8531	1.05	0.480
Pure Error	5	18.371	3.6742		
Total	19	199.035			

Table S3. Analysis of Variance for % antioxidant capacity

Source	DF	Adj SS	Adj MS	F-Value	P-value
Regression	9	0,002167	0,000241	2,14	0,126
Linear	3	0,001854	0,000618	5,48	0,017
time	1	0,001225	0,001225	10,86	0,008
Ratio S/S	1	0,000153	0,000153	1,36	0,270
Ratio E/W	1	0,000475	0,000475	4,22	0,067
Square	3	0,000188	0,000063	0,56	0,655
time*time	1	0,000162	0,000162	1,43	0,259
Ratio S/S*Ratio S/S	1	0,000035	0,000035	0,31	0,588
Ratio E/W*Ratio E/W	1	0,000000	0,000000	0,00	0,976
Interaction	3	0,000125	0,000042	0,37	0,777
time*Ratio S/S	1	0,000094	0,000094	0,84	0,382
time*Ratio E/W	1	0,000031	0,000031	0,27	0,614
Ratio S/S*Ratio E/W	1	0,000000	0,000000	0,00	0,967
Residual Error	10	0,001127	0,000113		
Lack-of-Fit	5	0,000429	0,000086	0,61	0,698
Pure Error	5	0,000699	0,000140		
Total	19	0,003294			
